# Supplementary material for: In Vitro Hepatic Models to Assess Herb–Drug Interactions: Approaches and Challenges
Source: Pharmaceuticals (Basel). 2023 Mar 8;16(3):409. doi: 10.3390/ph16030409 (PMC10058280; doi:10.3390/ph16030409)
Supplement: Supplementary file 1 [file pharmaceuticals-16-00409-s001.zip › pharmaceuticals-2234661-supplementary.pdf]

Table S1: Supplementary data

Liver Model	Number of Publications	Relevant references
Recombinant Enzymes	**83 946**	**https://doi.org/10.1080/0049825050015937**
Human liver microsomes (HLM)	**27 653**	**https://doi.org/10.5772/intechopen.108246**
S9 fractions	4790	**https://doi.org/10.1016/S0065-2490(05)80006-1**
Cytosolic Liver fractions	**4997**	https://doi.org/10.1042/bj2280363
**Precision Cut Liver slices**	1527	https://doi.org/10.1038/nprot.2010.111
**Primary Human Hepatocytes**	5 326	https://doi.org/10.2174/138920012802138589
**HepG2/C3A cells**	74	https://doi.org/10.1016/j.taap.2020.115279
